# The mosquito adulticidal *Chromobacterium* sp. Panama causes transgenerational impacts on fitness parameters and elicits xenobiotic gene responses

**DOI:** 10.1186/s13071-018-2822-8

**Published:** 2018-04-05

**Authors:** Sarah M. Short, Sarah van Tol, Brendan Smith, Yuemei Dong, George Dimopoulos

**Affiliations:** 1 W. Harry Feinstone Department of Molecular Microbiology and Immunology, Bloomberg School of Public Health, Johns Hopkins University, Baltimore, Maryland USA; 2 Microbiology and Immunology, University of Texas Medical Branch, Galveston, Texas USA

**Keywords:** *Anopheles gambiae*, *Chromobacterium*, Mosquito, Host-microbe interactions, Mosquitocide, Vector control, Transcriptome

## Abstract

**Background:**

Vector control is critical in reducing the disease burden caused by mosquitoes, and insecticides are an effective tool to control vector populations. Resistance to common insecticides is now widespread, and novel classes of insecticides are needed. In previous work, we described the mosquitocidal activity of *Chromobacterium* sp. Panama (*C.*sp_P), a bacterium found in association with mosquitoes in natural populations. In the current work, we further explored the effects of exposure to the bacterium on mosquito fitness and mosquito physiology.

**Results:**

We found that *C.*sp_P has mosquitocidal activity against a broad range of mosquito taxa. When exposed to *C.*sp_P as adults, female *An. gambiae* suffered reduced longevity, but experienced no change in fecundity. The offspring of these females, however, had higher mortality as larvae and were slower to develop compared to offspring of control females. We also found that the mosquitocidal activity of *C.*sp_P was retained after removal of live cells from biofilm culture media, suggesting the bacteria secrete mosquitocidal compound(s) into the media during growth. Exposure to this cell-free *C.*sp_P-conditioned media caused female midgut transcriptional changes comprising detoxification, xenobiotic response, and stress response genes, suggesting the physiological response to *C.*sp_P is similar to that of insecticide exposure. Finally, we found that multiple members of the *Chromobacterium* genus had mosquitocidal activity, but this activity was highest in mosquitoes treated with *C.*sp_P.

**Conclusions:**

Our findings suggest that *C.*sp_P produces factor(s) with strong effects on mosquito longevity and fitness, which may be of interest for mosquitocide development. More generally, they indicate that further exploration of mosquito-associated and environmental microbes for novel insecticidal compounds or biocontrol agents is warranted.

**Electronic supplementary material:**

The online version of this article (10.1186/s13071-018-2822-8) contains supplementary material, which is available to authorized users.

## Background

Vector-borne diseases such as malaria, dengue virus, and Zika virus represent a substantial public health burden, accounting for hundreds of millions of cases each year resulting in hundreds of thousands of deaths and severe sequelae in survivors [1–4]. One of the most effective ways of controlling pathogen transmission is vector control; in the case of malaria prevention this primarily involves the use of insecticide treated bed nets and indoor residual spraying [4, 5]. Resistance to common insecticides is a serious concern, and there remains a need for novel classes of insecticides that can be used to supplement the current repertoire [4, 6, 7]. Mosquito larvae develop in bacteria-rich pools of water and adults carry bacteria in their digestive tract, reproductive tract, and salivary glands throughout their lives [8–16]. These bacteria and other microbes represent a rich source of organisms that grow well in association with mosquitoes and, in addition to other environmental bacteria and fungi, may constitute a potential source of novel biocontrol and/or mosquitocidal agents.

In previous work, we described *Chromobacterium* sp. Panama (*C.*sp_P), isolated from the midguts of *Aedes aegypti* mosquitoes in Panama [17]. Bacteria from this genus are soil-dwelling, Gram-negative microbes, and are highly recognizable by the characteristic purple pigment called violacein produced by many members of the genus (though notably not by *C.*sp_P) [17, 18]. The most well-described member of the genus, *C. violaceum*, has been found to produce numerous bioactive factors with antimicrobial properties, as well as hydrogen cyanide, which can be used to “bio-leach” gold from discarded electronics [18, 19]. In our previous study, we found *C.*sp_P to have strong mosquitocidal activity against *Anopheles gambiae* (a primary vector of the malaria parasite *Plasmodium falciparum*) and *Aedes aegypti* (the primary vector of dengue and Zika viruses) adult females when exposed to the bacteria in a sugar meal [17]. Additionally, when present in the mosquito midgut, *C.*sp_P reduced susceptibility of *An. gambiae* and *Ae. aegypti* mosquitoes to *Plasmodium falciparum* and dengue virus, respectively [17]. These anti-pathogen properties are active *in vitro* (i.e. independent of the mosquito), suggesting the bacteria produces compound(s) with anti-pathogen activity [17]. Another member of the genus, *C. subtsugae*, has been shown to have insecticidal activity across diverse taxa of insects, including beetles, moths and whiteflies, though it has not been shown to be active against mosquitoes [20]. Additionally, a patent has been filed reporting that *C. vaccinii* is active against moths and *Ae. aegypti* mosquito larvae [21].

In the present study, we further explored the mosquitocidal properties of *C.*sp_P and its effects on mosquito fitness and mosquito physiology as measured by transcriptome changes upon exposure. We found that *C.*sp_P has broad insecticidal activity among mosquitoes, inducing mortality in three additional species of vector mosquitoes. We also found that *An. gambiae* females that survived exposure to *C.*sp_P suffered reduced fitness potential; their offspring had increased mortality as larvae and showed delayed time to pupation and eclosion. We determined that mosquitocidal factor(s) of *C.*sp_P persisted in cell-free preparations of bacteria cultured in biofilm conditions, and found that exposure to cell-free preparations of *C.*sp_P increased transcript abundance in genes related to detoxification and insecticide treatment and decreased transcript abundance of genes related to nucleosome and chromatin formation. Finally, we determined that other species in the *Chromobacterium* genus have mosquitocidal properties, suggesting production of insecticidal compounds is common throughout the genus.

## Methods

### Mosquito strains and maintenance

*Anopheles gambiae* (Keele strain), *An. stephensi* (Liston strain), *Ae. albopictus* (Gainsville strain, MRA-804 from BEI Resources), and *Culex quinquefasciatus* (JHB strain, NR-43025 from BEI Resources) were reared at 27 °C and 80% RH with a 14:10 light:dark photocycle.

### Bacterial information

*C.*sp_P was isolated from *Aedes aegypti* in Panama in 2010 [17, 22]. *Pantoea* sp. was isolated from *Anopheles arabiensis* in Zambia in 2010 [23]. *Chromobacterium violaceum* was obtained from ATCC (strain: ATCC 12472), and other species of *Chromobacterium* were obtained from the Leibniz Institute DSMZ: *C. aquaticum* (DSM 19852), *C. subtsugae* (DSM 17043) and *C. vaccinii* (DSM 25150).

### Bacterial culture growth

Bacteria were grown either in “planktonic” or “biofilm” conditions. To culture bacteria in planktonic conditions, we added 1 μl of pure freezer stock to 5 ml of LB and incubated with shaking at 30 °C for 16–18 h or 72 h, as indicated. The only exception to this protocol was for the experiment using multiple mosquito species, in which the planktonic culture was grown by inoculating 15 ml LB broth with 150 μl fresh culture and incubating at 30 °C for ~18 h with shaking. The fresh culture was grown by inoculating 5 ml LB with several single colonies grown from glycerol freezer stock and incubating overnight at 30 °C with shaking. To culture bacteria in biofilm conditions, we added 1 μl of pure freezer stock to 5 ml LB in a sterile 6-well cell culture plate and incubated without shaking at 30 °C for 72 h.

### Bacterial culture preparation

Planktonic cultures were pelleted and washed twice with 1× PBS then re-suspended in additional 1× PBS to 1.0 (± 0.1) OD_600_ and if necessary diluted or concentrated to achieve a desired cell density (as indicated). For the experiment using filtered planktonic culture, the culture was diluted to 1.0 (± 0.1) OD_600_ using sterile LB and not washed to preserve the supernatant. To collect biofilm, liquid media was removed from each well of the culture plate and 1 ml 1× PBS was added per well. Biofilm was removed from the culture plate by repeatedly pipetting the 1× PBS up and down in each well. Biofilm suspension was then transferred to a centrifuge tube and vortexed for 1 min to further encourage suspension in 1× PBS. To collect biofilm supernatant, liquid media was removed from biofilm culture wells, transferred to centrifuge tubes and vortexed for 1 min. To filter each preparation, cultures were pelleted at 5000 *rpm* for 3–5 min and the liquid supernatants were passed through a 0.2 μm syringe filter.

To measure CFU/ml of each bacterial culture preparation, an aliquot of unfiltered culture was serially diluted in 1× PBS and plated on LB agar. Colony forming units were counted from each dilution that yielded countable colonies and averaged to calculate CFU/ml. For *C.*sp_P, diluting overnight planktonic culture to 1.0 OD_600_ resulted in an average of 4.59 × 10^8^ CFU/ml (range of CFU/ml measurements: 1.25 × 10^7^ to 9.75 × 10^8^). When a higher or lower cell density was needed, CFU/ml was determined *post-hoc*, and in those instances CFU/ml is indicated in the respective figure legend. On average, *C.*sp_P biofilm suspension contained 5.03 × 10^8^ CFU/ml (range of CFU/ml measurements: 1.30 × 10^8^ to 1.42 × 10^9^), and biofilm supernatant contained 8.60 × 10^7^ CFU/ml (range of CFU/ml measurements: 5.0 × 10^6^ to 1.45 × 10^8^).

To test whether the mosquitocidal factor contained in the *C.*sp_P supernatant was volatile, *C.*sp_P biofilm supernatant and sterile LB were filter sterilized and aliquoted into 1.5 ml microcentrifuge tubes and the open tubes were centrifuged for 30 min under a vacuum in a Vacufuge (Eppendorf, Hamburg, Germany). Non-centrifuged controls for both sample types were maintained at room temperature with lids sealed. Lost volume of vacuum centrifuged samples was reconstituted by adding sterile water to the centrifuged samples to the same final volume as samples that were not centrifuged. All four preparations were fed to *An. gambiae* females in sucrose meals as described below.

### Measuring short-term survival in adults after bacterial exposure

Adult females were cold anaesthetized 3–7 days after eclosion, sorted into cardboard cups, and provided 10% sucrose *ad libitum* until the experiment. Mosquitoes were starved overnight to encourage feeding, and the morning of the experiment cultures were harvested and prepared as described above. Bacterial culture preparations were added to sterile sucrose, and 1× PBS or LB media were added in place of bacteria as controls. Unless otherwise stated, bacterial preparations (unfiltered and filtered), PBS and LB were mixed 1:1 with 3% sucrose, and in all cases the final sucrose concentration was 1.5%. All sugar meals were provided to females for 24 h in microcentrifuge tubes containing sterile filter paper wicks. Experimental sugar meals were then removed, and all treatments were provided 10% sucrose *ad libitum*. Survival was monitored for seven to ten days after treatment commenced.

### Blood-feeding

Blood meals were prepared by mixing 40% human red blood cells and 60% heat inactivated human serum. Mosquitoes were starved 6–8 h before being allowed to blood-feed for 1 h using membrane feeders as described previously [24]. After feeding, females were cold anaesthetized and unfed females were removed from the experiment.

### Fecundity and oviposition experiments

Six-to seven-day old females were fed a 10^7^ dose of *C.*sp_P or *P.* sp, or PBS as a control, for 24 h and each treatment was blood-fed approximately 56 h post-exposure. This dose of *C.*sp_P was chosen because it is high enough to ensure efficient exposure and moderate mortality in *An. gambiae*, but low enough that sufficient numbers of females survive through completion of the experiment. Females that did not blood-feed were removed from the experiment. Three days later, blood-fed females were placed into individual oviposition cups (50 ml conical bottom tubes containing 5–7.5 ml of deionized water and a piece of filter paper). After being allowed to oviposit for two days, the number of ovipositing females and the number of eggs oviposited were recorded. If females oviposited ten or fewer eggs, insemination was verified by spermatheca dissection. The spermathecae were dissected in 30 μl of 1× PBS then transferred to 10 μl of Geimsa stain (diluted 1:100 in deionized water) and physically crushed onto a glass slide using ethanol-sterilized forceps, allowed to air dry, fixed with 50 μl of methanol for 30 min, and rinsed with deionized water. Slides were viewed using a light microscope at 10–40×. Only inseminated females were included in the oviposition and fecundity analyses, and rate of insemination did not significantly differ between the treatment groups (*χ*^2^ = 4.77, *df* = 2, *P* = 0.092, Additional file 1: Figure S1).

### Development and life history experiments

Adult *An. gambiae* females were fed a 1.5% sucrose solution containing *C.*sp_P at a cell density of 10^7^ CFU/ml or 1× PBS as a control for 24 h. Approximately two days after *C.*sp_P exposure, females were then given a blood meal and allowed to oviposit. Eggs were hatched and 100 larvae per treatment were transferred to clean trays. Larval survival and pupation events were recorded. Three 1 ml samples of water were collected from the oviposition water, larval trays before adding the food and larvae, and larval trays at 4 and 8 days after transferring larvae to the trays. Water samples were plated onto LB agar plates at 10-fold serial dilutions to calculate CFU, and unique colonies were isolated and identified to bacterial species using the *16S* rDNA sequence [25]. Pupae were then transferred to a small beaker in a cage and pupal survival and eclosion events were monitored. Upon eclosion, male and female adults were transferred to separate cups (18–23 individuals per cup) and survival was monitored until all adults were dead.

### Microarray experimental design, sample preparation and analysis

Six to seven day old adult *An. gambiae* females were starved overnight and provided sugar meals containing 3% sucrose mixed 1:1 with either LB or 72 h *C.*sp_P cell-free biofilm supernatant (final sucrose concentration 1.5%). Both LB and *C.*sp_P supernatant were filter sterilized before being added to the sucrose solution. After 24 h, midguts were dissected from 20 adult females per treatment in sterile 1× PBS on a cold block and pooled in TRIzol (Invitrogen, Carlsbad, USA) reagent on ice. Midguts were stored at -80 °C until RNA extraction. The experiment was repeated four independent times, with one pool of 20 midguts collected per treatment per replicate. RNA was extracted following the manufacturer’s protocol and genomic DNA was removed using the TURBO DNA-*free* Kit (Invitrogen) according to the manufacturer’s instructions. Quality of RNA was verified using an Agilent Bioanalyzer 2100. Transcriptome analysis was performed using a custom Agilent microarray described previously [26, 27]. All samples were labeled using the Two-Color Low Input Quick Amp Labeling Kit (Agilent Technologies, Santa Clara, USA) according to the manufacturer’s instructions. 200 ng of RNA from each sample was used as input for the labeling reaction and labeled cRNA was purified using the RNeasy Mini Kit (Qiagen, Hilden, Germany). Hybridization was performed according to Agilent’s Two-Color Microarray-Based Gene Expression Analysis Protocol. RNA extracted from LB-fed individuals was hybridized to that of individuals fed cell-free *C.*sp_P biofilm supernatant collected in the same replicate experiment, and samples were labeled in a dye-swap design to prevent dye bias. Feature extraction was performed using an Agilent Scanner and Agilent Feature Extraction Software. Analysis of microarray data was performed as in [28]. In brief, differential transcript abundance between LB-fed and *C.*sp_P-fed female midguts was assessed using limma in R [29] after background correction using the “normexp” method [30] and after normalizing signals using global loess within array normalization [31]. Lists of genes with differential transcript abundance between treatments were then assessed for Gene Ontology term enrichment using DAVID [32, 33].

### Statistical analysis

Unless otherwise stated, Cox proportional hazards models were used to assess the effect of treatment on survival. Individuals that were excluded during the experiment or were still alive at the conclusion of the experiment were treated as censored data. For all analyses, experimental replicate was included as a co-factor to account for variation across replicate experiments. For the experiments testing the effect of multiple species of *Chromobacterium* on survival and the effect of *Chromobacterium* sp. Panama and *Pantoea* sp. on survival, the data did not meet the assumptions of a proportional hazards model and therefore pairwise Log-Rank tests followed by a multiple testing correction were used. For the experiments testing survival of offspring and pupation/eclosion rates, Log-Rank tests were performed. All Cox proportional hazards and Log-Rank tests were performed in R [34]. Fecundity and oviposition data were analyzed by Kruskal-Wallis test in R and one-way ANOVA in GraphPad Prism. Larval bacterial load data and bacterial load data from biofilm *versus* supernatant were analyzed by two-way ANOVA in R.

## Results

We have shown in previous work that ingestion of *Chromobacterium* sp. Panama (*C.*sp_P) causes reduced longevity in adult *An. gambiae* and *Ae. aegypti* mosquitoes [17]. We were interested in further exploring the effects of *C.*sp_P on mosquito fitness and its adulticidal activity. For this, we investigated how exposure to *C.*sp_P during adulthood influences fecundity of *An. gambiae* females as well as development rate and survival of their offspring. We then probed the nature of the adulticidal activity of *C.*sp_P, and the effects of compounds secreted by *C.*sp_P on the transcriptome of adult female *An. gambiae*. We also explored other members of the genus *Chromobacterium* to determine whether species related to *C.*sp_P display adulticidal activity as well.

### *C.*sp_P has adulticidal effects against a broad range of disease vector mosquitoes

**Fig. 1:**
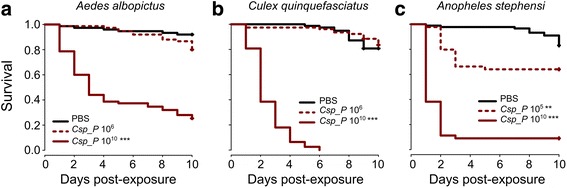
*C.*sp_P exposure causes mortality across a broad taxonomic range of disease vector mosquitoes. Planktonic overnight cultures of *C.*sp_P were washed two times with 1× PBS and diluted or concentrated to obtain “low” (i.e. approximately 10^5^ or 10^6^ CFU/ml) and “high” (i.e. approximately 10^10^ CFU/ml) bacterial cell densities, as per our previous work [17]. Bacteria of each density or 1× PBS as a control were then mixed 1:1 with 3% sucrose (final sucrose concentration 1.5%) and provided to adult females for 24 h, at which point all treatments were given 10% sucrose. **a** *Aedes albopictus* females: PBS *vs C.*sp_P 10^6^, *z* = 1.93, *P* = 0.0531; PBS *vs C.*sp_P 10^10^, *z* = 6.67 *P* < 0.0001. **b** *Culex quinquefasciatus*: PBS *vs C.*sp_P 10^6^, *z* = -1.06, *P* = 0.289; PBS *vs C.*sp_P 10^10^, *z* = 9.30, *P* < 0.0001. **c** *Anopheles stephensi*: PBS *vs C.*sp_P 10^5^, *z* = 2.87, *P* = 0.004; PBS *vs C.*sp_P 10^10^, *z* = 10.26, *P* < 0.0001. **a** and **c** were repeated 3 times with 25–30 individuals per replicate, while **b** was repeated 5 times with 9–20 individuals per replicate. Survival curves were fitted using the Kaplan-Meier method with pooled data from all replicates. Vertical tick-marks indicate censored samples. Data from all experiments were analyzed using a Cox proportional hazards model including treatment and replicate

In previous work, we showed that oral exposure to *C.*sp_P caused reduced longevity in *An. gambiae* and *Ae. aegypti* mosquitoes. We investigated the effects of oral exposure to *C.*sp_P on three additional species of disease vector mosquitoes: *Ae. albopictus*, *Culex quinquefasciatus* and *An. stephensi*. We allowed adult females to feed on two different densities of *C.*sp_P, or PBS as a control, in a sugar meal for 24 h and monitored survival for ten days post-exposure. We found that *An. stephensi* showed significantly reduced survival after exposure to *C.*sp_P at both a lower (10^5^ CFU/ml) and a higher (10^10^ CFU/ml) bacterial cell density, while the survival of *Ae. albopictus* and *C. quinquefasciatus* was significantly reduced only after exposure to *C.*sp_P at a density of 10^10^ CFU/ml (Fig. 1).

### *C.*sp_P exposure has no effect on fecundity among surviving females but reduces larval survival and slows development of their offspring

**Fig. 2:**
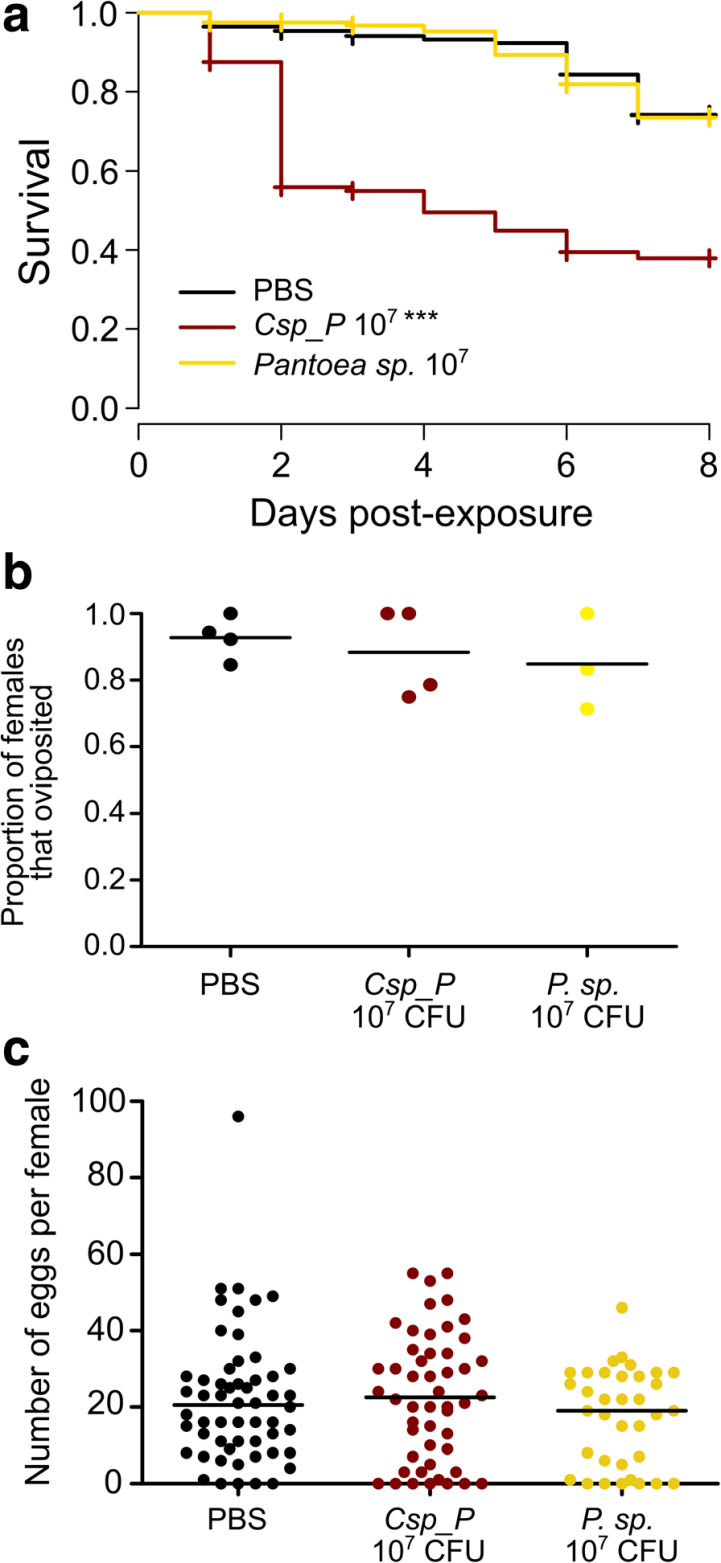
*C.*sp_P exposure causes adult mortality but does not influence fecundity of surviving females. Female *An. gambiae* mosquitoes were fed a 10^7^ CFU/ml dose of either *C.*sp_P or *Pantoea* sp. (*P.* sp.) planktonic culture or an equal volume of 1× PBS in a 1.5% sucrose meal for 24 h. Experimental sugar meals were then removed, and all treatments were provided 10% sucrose *ad libitum*. Females were blood-fed on the third day post-exposure and allowed to oviposit for 2 days. Females that did not blood-feed were removed from the experiment. Data for each treatment were collected over 3–4 biological replicates. **a** Exposure to *C.*sp_P 10^7^ CFU/ml significantly decreased survival relative to PBS, while exposure to *Pantoea* sp. did not have a significant effect on survival (PBS *vs C.*sp_P 10^7^, *χ*^2^ = 98.7, *df* = 1, *P* < 0.0001; PBS *vs Pantoea* 10^7^, *χ*^2^ = 0.1, *df* = 1, *P* = 0.77). Total sample sizes for **a**: *n*_PBS_ = 283, *n*_Pantoea 10^7_ = 162, *n*_*C.*sp_P 10^7_ = 264. **b**, **c** Treatment did not significantly affect the proportion of females that oviposited (**b**, *χ*^2^ = 0.68, *df* = 2, *P* = 0.7116) nor their fecundity (**c**, *F*_(2, 138)_ = 1.165, *df* = 2, *P* = 0.1474). Total sample sizes for **b** and **c**: *n*_PBS_ = 56, *n*_*Pantoea* 10^7_ = 35, *n*_*C.*sp_P 10^7_ = 50. Data were analyzed using pairwise Log-Rank tests in **a**, Kruskal-Wallis test in **b** and a one-way ANOVA in **c**. Black bars represent the median value in (**b**) and (**c**)

*C.*sp_P exposure causes significant mortality among adult *An. gambiae* females, but we were interested in determining whether females that survive suffer residual effects on fitness traits. We exposed adult *An. gambiae* females to *C.*sp_P in a 1.5% sugar meal at a density of 10^7^ CFU/ml, which causes moderate mortality (Fig. 2a). Simultaneously, we exposed adult *An. gambiae* to *Pantoea* sp. bacteria which does not cause mortality at the same cell density (Fig. 2a), to control for the potential nutritional impact of bacterial ingestion on egg production. We then blood-fed females from all treatments and assessed probability of oviposition and average total number of eggs laid per female. We found that *C.*sp_P exposure had no effect on probability of oviposition or number of eggs laid relative to either the PBS- or *Pantoea* sp.-exposed controls (Fig. 2b, c).

**Fig. 3:**
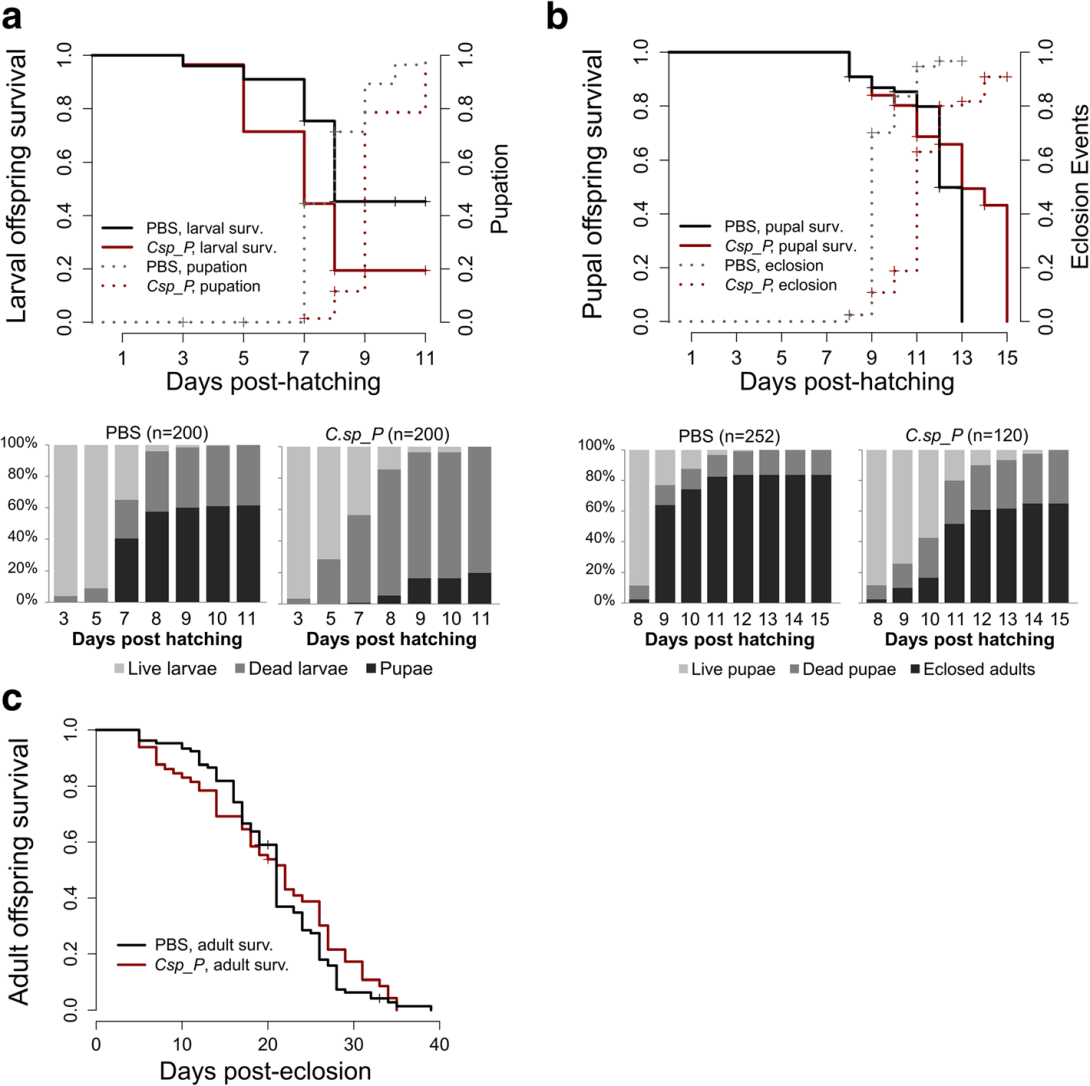
Exposure of adult females to *C.*sp_P causes increased larval mortality and slows rate of development in offspring. Adult female *A. gambiae* mosquitoes were fed a 10^7^ CFU/ml dose of planktonic *C.*sp_P or an equal volume of PBS in a 1.5% sucrose meal. Two days later, they were given a blood meal and allowed to oviposit. Eggs were collected and hatched, and survival and development time of offspring was monitored. **a** The survival of larval offspring of *C.*sp_P-exposed females was reduced significantly relative to larval offspring of control females (*χ*^2^ = 43.1, *df* = 1, *P* < 0.0001), and time to pupation was lengthened as well (*χ*^2^ = 101, *df* =1, *P* < 0.0001). Maternal *C.*sp_P exposure extended the median time to pupation by 24 h (PBS median time to pupation = 8 days, *C.*sp_P median time to pupation = 9 days). This experiment was repeated twice; total sample sizes were *n*_PBS_ = 200, *n*_*C.*sp_P_ = 200. **b** The survival of pupal offspring of *C.*sp_P-exposed females was not significantly different from that of pupal offspring of control females (*χ*^2^ = 0.2, *df* = 1, *P* = 0.67). However, time to eclosion was significantly lengthened in offspring of *C.*sp_P-treated mothers (*χ*^2^ = 103, *df* = 1, *P* < 0.0001). Maternal *C.*sp_P exposure extended the median time to eclosion by 48 h (PBS median time to eclosion = 9 days, *C.*sp_P median time to eclosion = 11 days). This experiment was repeated four times; total sample sizes were *n*_PBS_ = 252, *n*_*C.*sp_P_ = 120. **c** The longevity of adult offspring of *C.*sp_P-exposed females did not differ significantly from that of adult offspring of control females (*z* = 1.073, *P* = 0.28). This experiment was repeated three times; total sample sizes were *n*_PBS_ = 105, *n*_*C.*sp_P_ = 65. Data were analyzed using Log-Rank tests (for **a** and **b**) and Cox proportional hazards model including treatment and replicate (for **c**)

In a subsequent experiment, we exposed adult female mosquitoes to *C.*sp_P in a 1.5% sugar meal at a density of 10^7^ CFU/ml and then allowed them to blood-feed and lay eggs. We recorded the rate of development and monitored survival of the offspring across all stages of development. We found that the proportion of eggs that hatched was not significantly different between treatments; mean hatch rate for PBS was 0.612 (95% CI: 0.75–0.48), while for *C.*sp_P it was 0.628 (95% CI: 0.73–0.53, *t* = 0.902, *df* = 4, *P* = 0.418). However, larval mortality was significantly higher for offspring of *C.*sp_P-exposed mothers compared to offspring of PBS-exposed mothers (Fig. 3a, *χ*^2^ = 43.1, *df* = 1, *P* < 0.0001). Additionally, the rate of pupation was significantly slower for offspring of *C.*sp_P-exposed mothers relative to that of PBS-exposed mothers; the median time to pupation was nine days and eight days, respectively (Fig. 3a, *χ*^2^ = 101, *df* =1, *P* < 0.0001). Survival was not significantly different between the groups at the pupal stage, however, time to eclosion was significantly longer in offspring of *C.*sp_P-exposed mothers (median time to eclosion = 11 days) compared to offspring of PBS-exposed mothers (median time to eclosion = 9 days) (Fig. 3b, *χ*^2^ = 103, *df* = 1, *P* < 0.0001). Survival of F1 adults was similar between the two groups (Fig. 3c). We sampled oviposition water and found live *C.*sp_P in 0 of 9 samples collected over three replicates. We also sampled larval water at days 0, 4, and 8 post-larval transfer and found live *C.*sp_P in one sample taken from a single replicate on day four. All other larval water samples were negative for *C.*sp_P. In addition to testing for *C.*sp_P in these water samples, we also quantified CFU/ml of all bacteria that grew on LB agar from each water sample and found that overall bacterial load did not significantly differ between treatments (Additional file 2: Figure S2).

### *C.*sp_P mosquitocidal activity persists after removal of live bacteria when grown in biofilm but not planktonic conditions

**Fig. 4:**
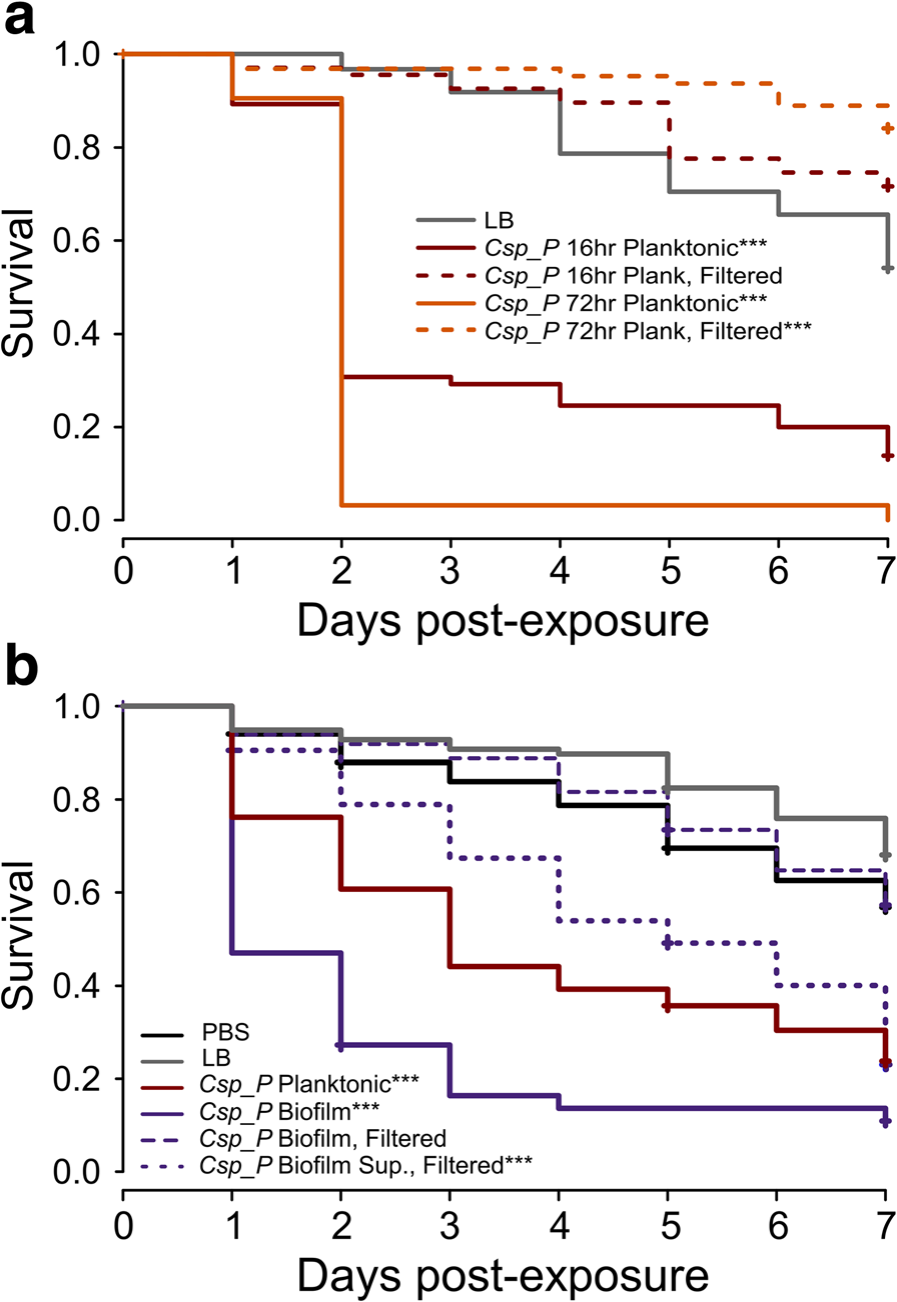
Persistence of adulticidal activity in cell-free preparations of *C.*sp_P. **a** *C.*sp_P adulticidal activity is not retained after removal of live cells from planktonic culture. *C.*sp_P was grown at 30 °C with shaking for approximately 16 or 72 h and diluted to 1.0 (± 0.1) OD_600_ with additional sterile LB. An aliquot of each culture was then filtered through a 0.2 μm filter to remove live bacterial cells. Each culture preparation or LB as a control was mixed 1:1 with 3% sucrose (1.5% sucrose final concentration) and provided to *An. gambiae* adult females for 24 h. Experimental sugar meals were then removed, and all treatments were provided 10% sucrose *ad libitum*. Survival was monitored for seven days. The experiment was repeated three independent times, with 17–25 individuals per treatment per replicate. Survival of each treatment was compared to the LB control using a Cox proportional hazards model: LB *vs C.*sp_P 16hr Plank Unfilt, *z* = 7.11, *P* < 0.0001; LB *vs C.*sp_P 16hr Plank Filt, *z* = -1.76, *P* = 0.078; LB *vs C.*sp_P 72hr Plank Unfilt, *z* = 9.57, *P* < 0.0001; LB *vs C.*sp_P 72hr Plank Filt, *z* = -3.25, *P* = 0.001. **b** *C.*sp_P adulticidal activity is retained after removal of live cells from biofilm culture media. *C.*sp_P was grown in biofilm conditions for 72 h at which point the biofilm and the LB media overlaying the biofilm (i.e. biofilm supernatant) were collected. Both biofilm and supernatant were filtered through a 0.2 μm filter to remove live bacterial cells. Each culture preparation, LB or PBS were mixed 1:1 with 3% sucrose, provided to *An. gambiae* adult females for 24 h and mortality was monitored for 7 days. Control for all samples is PBS with the exception of *C.*sp_P biofilm supernatant, for which the control is LB. All treatments caused a significant reduction in survival except filtered *C.*sp_P biofilm: PBS *vs C.*sp_P Planktonic, *z* = 6.13, *P* < 0.0001; PBS *vs C.*sp_P Biofilm, *z* = 11.34, *P* < 0.0001; PBS *vs C.*sp_P Biofilm Filtered, *z* = -0.33, *P* = 0.745; LB *vs C.*sp_P Biofilm Sup. Filtered, *z* = 6.66, *P* < 0.0001. The entire experiment was repeated four independent times with 18–40 individuals per treatment per replicate, and the data were analyzed using a Cox proportional hazards model

We were interested in further exploring the nature of the mosquitocidal compound(s) produced by *C.*sp_P to better understand the mechanism by which it causes mosquito mortality. Because treatment with live bacteria introduces substantial variability and potential for dynamic rates of exposure over time, we tested whether *C.*sp_P secretes the mosquitocidal activity(ies) into the culture media by assaying whether cell-free preparations exerted mosquitocidal activity. We grew *C.*sp_P in planktonic culture for approximately 16 h or 72 h and filtered live bacteria from an unwashed aliquot of each culture. We then provided *An. gambiae* females with 1.5% sugar meals containing filtered and unfiltered preparations from each incubation time or LB as a control. We found that both unfiltered cultures caused significant mortality over seven days compared to LB (Fig. 4a; *C.*sp_P_16hr Unfilt_, *z* = 7.11, *P* < 0.0001; *C.*sp_P _72hr Unfilt_, *z* = 9.57, *P* < 0.0001), but that when live bacteria were filtered out of the culture, survival was either no different or significantly better than the control (Fig. 4a; *C.*sp_P _16hr Filt_, *z* = -1.76, *P* = 0.078; *C.*sp_P _72hr Filt_, *z* = -3.25, *P* = 0.001).

We also grew *C.*sp_P in biofilm conditions (30 °C, without shaking) for 72 h and investigated whether adulticidal activity is present under these growth conditions and whether it persists after removal of live bacterial cells. We found that *An. gambiae* females given *C.*sp_P biofilm re-suspended in a sugar meal had significantly reduced survival relative to the PBS control (Fig. 4b, *P* = 2.0 × 10^-16^). We also filtered the biofilm resuspension as well as the biofilm supernatant (i.e. the LB media in which the biofilm grew) to remove live cells and exposed *An. gambiae* females to sugar meals containing each cell-free preparation. We found that filtering eliminated adulticidal activity from *C.*sp_P biofilm, but not from *C.*sp_P biofilm supernatant when compared to LB (Fig. 4b, PBS *vs C.*sp_P_Biofilm Filt,_ *z* = -0.33, *P* = 0.745, LB *vs C.*sp_P_Biofilm Sup. Filt_, *z* = 6.66, *P* < 0.0001).

### *C.*sp_P –produced hydrogen cyanide does not mediate the adulticidal activity

**Fig. 5:**
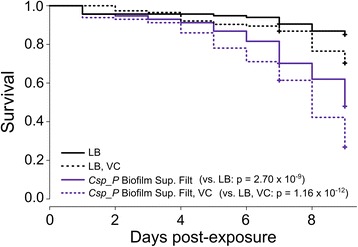
*C.*sp_P-produced adulticidal factor is non-volatile. Filtered (i.e. cell-free) *C.*sp_P biofilm supernatant and LB were spun in open containers in a vacuum centrifuge for 30 min at room temperature to allow for evaporation of volatile compounds. Original volume of vacuum centrifuged (VC) samples was then reconstituted using sterile water. Cell-free *C.*sp_P biofilm supernatant (VC and not) or LB as a control (VC and not) were mixed 1:1 with 3% sucrose (1.5% final sucrose concentration) and fed to *An. gambiae* females for 24 h. Experimental sugar meals were then removed and survival was monitored daily. The experiment was repeated four independent times, with 26–30 individuals per treatment per replicate. Survival analysis was performed using a Cox proportional hazards model testing the effect of bacterial treatment (*C.*sp_P *vs* LB), vacuum centrifugation, and an interaction between the two factors. No interaction was detected, indicating that the effect of *C.*sp_P ingestion was consistent regardless of whether samples were vacuum centrifuged. Main effects of both bacterial treatment (*z* = 9.15, *P* < 0.0001) and vacuum centrifugation (*z* = 4.08, *P* < 0.0001) were significant. In pairwise comparisons between their respective LB controls, *C.*sp_P (*z* = 5.95, *P* < 0.0001) and *C.*sp_P VC (*z* = 7.11, *P* < 0.0001) treatments had highly significant effects on survival

Hydrogen cyanide (HCN) is a known secondary metabolite of multiple *Chromobacterium* species [21, 35]. HCN is a generalist poison and is therefore one potential source of mosquitocidal activity of *C.*sp_P against adults. We tested cell-free *C.*sp_P biofilm supernatant for hydrogen cyanide and found the average concentration to be 0.039 mg/l (range: 0.023–0.047 mg/l). The concentration of HCN in the sugar meals fed to adults in our experiments is therefore approximately 0.02 μg HCN/ml. Hydrogen cyanide is a gas and boils at 25.6 °C. We therefore hypothesized that if HCN is causing adult mosquito mortality, vacuum centrifugation would eliminate the mosquitocidal effect due to evaporation of HCN. We centrifuged *C.*sp_P filtered biofilm under a vacuum in open microcentrifuge tubes to allow evaporation and tested the impact of vacuum centrifugation on mosquitocidal activity. We found that exposure to *C.*sp_P biofilm supernatant reduced survival regardless of whether the sample had been vacuum centrifuged (Fig. 5, *C.*sp_P *vs* LB, *z* = 5.95, *P* < 0.0001; *C.*sp_P_V.C._ *vs* LB_V.C._, *z* = 7.11, *P* < 0.0001). Vacuum centrifugation also reduced survival, and this was consistent across the experimental and control treatments (Fig. 5, *z* = 4.08, *P* < 0.0001).

### Exposure to *C.*sp_P filtered biofilm supernatant alters transcript abundance in genes important for detoxification, insecticide resistance, and stress response

**Fig. 6:**
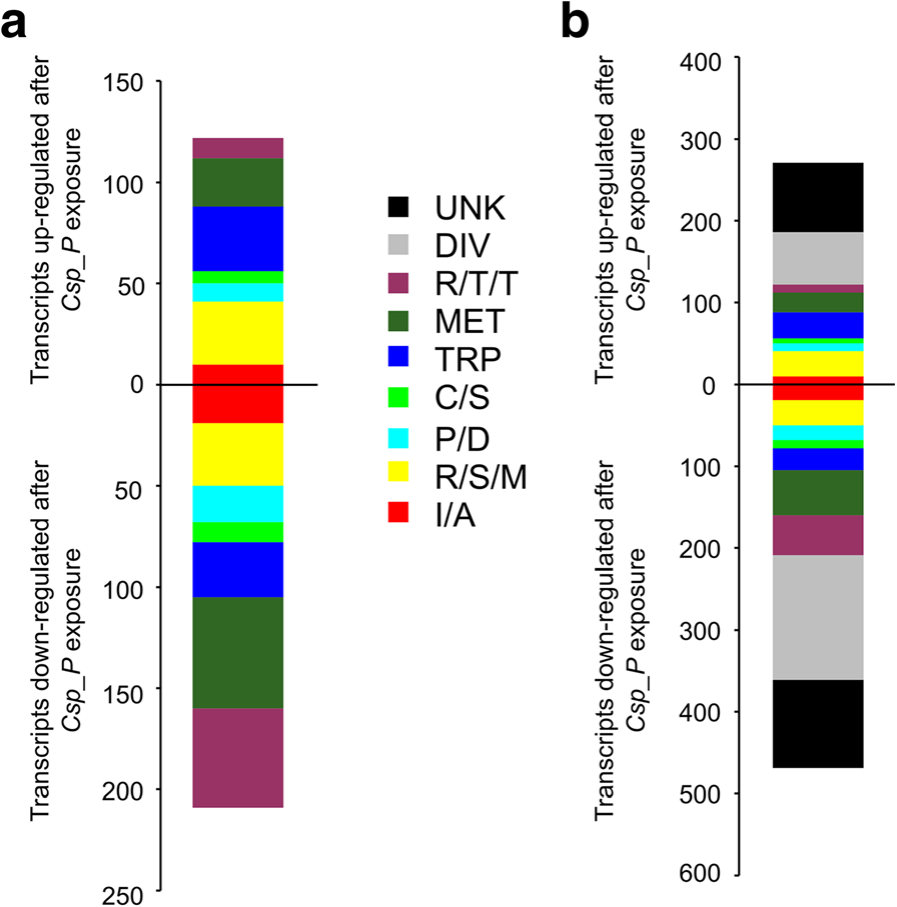
Effect of exposure to cell-free *C.*sp_P biofilm supernatant on transcript abundance in the midguts of adult female *An. gambiae*. Adult *An. gambiae* females were fed sugar meals containing either LB or 72 h filter-sterilized *C.*sp_P biofilm supernatant for 24 h, at which point midguts were dissected from 20 adult females per treatment in sterile 1× PBS and pooled in TRIzol. RNA from LB-fed females was co-hybridized with RNA from *C.*sp_P-fed females to a custom Agilent microarray. The experiment was repeated in full four independent times, and samples were co-hybridized within the same replicate experiment. The graph shows the number of genes from each functional category where transcript abundance significantly increased or decreased in response to cell-free *C.*sp_P biofilm supernatant exposure. **a** Genes from known functional categories. **b** Including genes with unknown function and diverse function. *Abbreviations*: UNK, unknown; DIV, diverse; R/T/T, replication, transcription, and translation; MET, metabolism; TRP, transport; C/S, cytoskeletal and structural; P/D, proteolysis and digestion; R/S/M, redox, stress-related, and mitochondrial; I/A, immunity and apoptosis

To gain insight on how *C.*sp_P affects adult females, we performed a genome-wide transcriptome analysis comparing transcript abundance of females fed a sugar meal containing filtered *C.*sp_P (i.e. cell free) biofilm supernatant *versus* LB as a control. Transcript abundance was analyzed in midgut tissues harvested 24 h after introduction of experimental sugar meals. We found that exposure to cell-free *C.*sp_P biofilm supernatant caused altered transcript abundance of 62 genes involved in stress response (R/S/M), 79 involved in metabolism (MET) and 59 involved in replication, transcription, and translation (R/T/T) (Fig. 6, Additional file 3). A gene ontology enrichment analysis confirmed these observations. Genes significantly up- or downregulated by cell-free *C.*sp_P biofilm supernatant exposure were enriched for Biological Process GO terms related to “response to xenobiotic stimulus” and “response to insecticide” (Table 1). This was primarily driven by the upregulation of multiple *cytochrome P450* genes; 14 members of the CYP6 subfamily were significantly upregulated in response to *C.*sp_P treatment, as were members of the CYP4 and CYP9 subfamilies (Additional file 3). Other Biological Process GO terms that were significantly enriched include those related to “organic acid metabolic process.” Cellular Component GO terms significantly overrepresented included those related to “chromatin,” “DNA packaging complex,” and “protein DNA complex,” a result driven by the downregulation of multiple genes coding for histone proteins and DNA repair proteins (Table 1, Additional file 3).

**Table 1:** Gene Ontology term enrichment among genes whose transcripts are affected by exposure to *C.*sp_P cell-free biofilm supernatant

GO term ID	GO term name	Count	Fold enrichment	BH corrected *P-*value
Biological process				
GO:0044712	Single-organism catabolic process	30	2.35	3.72E-03
GO:0043436	Oxoacid metabolic process	39	2.13	3.85E-03
GO:0042178	Xenobiotic catabolic process	10	6.20	4.30E-03
GO:0071466	Cellular response to xenobiotic stimulus	10	6.20	4.30E-03
GO:0006805	Xenobiotic metabolic process	10	6.20	4.30E-03
GO:0019752	Carboxylic acid metabolic process	38	2.09	4.33E-03
GO:0006082	Organic acid metabolic process	40	2.00	4.81E-03
GO:0009410	Response to xenobiotic stimulus	10	5.94	5.17E-03
GO:0009407	Toxin catabolic process	10	6.48	5.57E-03
GO:0090487	Secondary metabolite catabolic process	10	6.48	5.57E-03
GO:0017085	Response to insecticide	10	6.48	5.57E-03
GO:0009404	Toxin metabolic process	10	6.48	5.57E-03
GO:0017143	Insecticide metabolic process	10	6.48	5.57E-03
GO:0046701	Insecticide catabolic process	10	6.48	5.57E-03
GO:0046680	Response to DDT	10	6.48	5.57E-03
GO:0009636	Response to toxic substance	11	5.81	9.92E-03
GO:0006334	Nucleosome assembly	10	4.32	4.72E-02
Molecular function				
GO:0016705	Oxidoreductase activity, acting on paired donors, with incorporation or reduction of molecular oxygen	25	2.45	2.08E-02
GO:0004497	Monooxygenase activity	21	2.62	2.47E-02
GO:0005506	Iron ion binding	25	2.50	3.08E-02
GO:0046906	Tetrapyrrole binding	22	2.34	4.92E-02
GO:0020037	Heme binding	22	2.35	5.60E-02
Cellular component				
GO:0032993	Protein-DNA complex	20	6.01	2.02E-08
GO:0000786	Nucleosome	16	6.45	4.20E-07
GO:0044815	DNA packaging complex	16	5.65	2.52E-06
GO:0000785	Chromatin	20	3.98	1.35E-05
GO:0000790	Nuclear chromatin	13	5.40	8.46E-05
GO:0005694	Chromosome	29	2.48	3.21E-04
GO:0000788	Nuclear nucleosome	6	14.12	3.70E-04
GO:0000228	Nuclear chromosome	17	3.20	1.50E-03
GO:0044427	Chromosomal part	25	2.42	1.75E-03
GO:0044454	Nuclear chromosome part	15	3.03	7.53E-03
GO:0005811	Lipid particle	6	7.70	1.34E-02

### Oral exposure to multiple *Chromobacterium* species causes mortality in adult mosquitoes

**Fig. 7:**
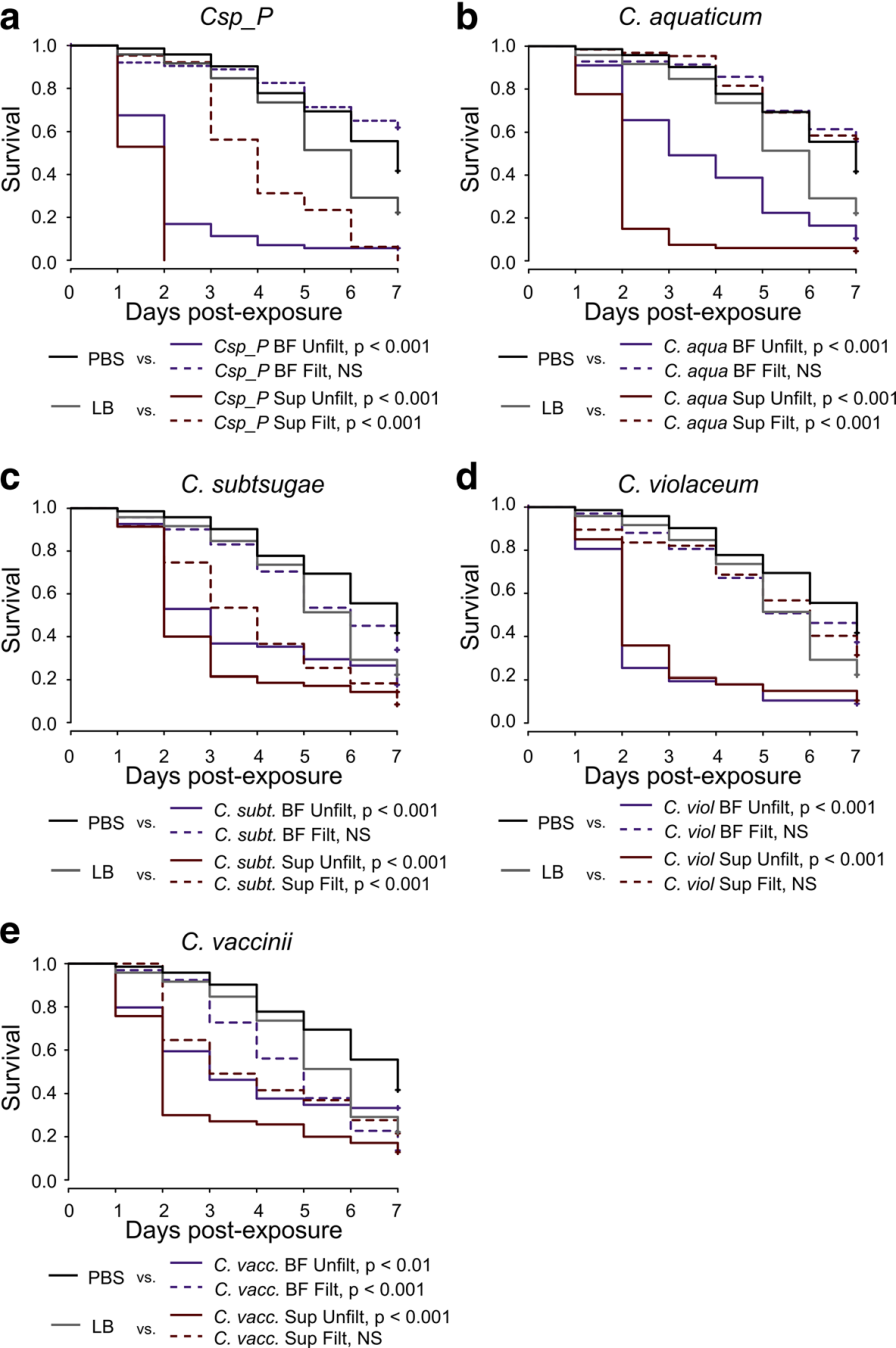
Exposure to multiple *Chromobacterium* species affects mortality of *An. gambiae* females. Five *Chromobacterium* species were cultured under biofilm conditions for 72 h and the biofilm and culture media (i.e. biofilm supernatant) were harvested and an aliquot was filtered through a 0.2 μm filter to remove live cells. Culture preparations, LB or PBS were mixed 1:1 with 3% sucrose and provided to *An. gambiae* adult females for 24 h. Experimental sugar meals were then removed, and all treatments were provided 10% sucrose *ad libitum*. Survival was monitored for seven days. The experiment was repeated three independent times, with 20–25 individuals per treatment per replicate. Only one PBS and LB control dataset was collected, and these data are repeated on each panel to allow visualization of each individual species. Contrasts between each treatment and the appropriate control (PBS for biofilm, LB for biofilm supernatant) were conducted using Log-Rank tests followed by a Bonferroni correction

At least two species of *Chromobacterium*, *C. subtsugae* and *C. vaccinii,* have been shown to have insecticidal properties, and *C. subtsugae* is currently being used as a biocontrol agent marketed as Grandevo® (Marrone BioInnovations) [20, 21, 36]. *Chromobacterium vaccinii* causes mortality of *Aedes aegypti* larvae when added to the larval breeding water [21]. *Chromobacterium subtsugae* was shown to cause mortality in diverse insect taxa, though not in the larvae of *Culex pipiens*, the only mosquito species on which it was tested [20]. We investigated whether other species of *Chromobacterium* in addition to *C.*sp_P induce mortality in *An. gambiae* adults and whether the mosquitocidal activity is maintained after removal of live cells, as it is in *C.*sp_P. To test this, we grew five bacterial species in biofilm conditions for 72 h: *C.*sp_P, *C. aquaticum*, *C. subtsugae*, *C. violaceum* and *C. vaccinii*. We then isolated the biofilm as well as the surrounding media (biofilm supernatant) from each species and provided filtered (i.e. cell-free) and unfiltered preparations to adult *An. gambiae* females in sugar meals for 24 h. CFUs per ml were similar among the species for each fraction, but across all species, the biofilm fraction harbored more CFUs than the supernatant (*F*_(1, 22)_ = 11.26, *df* = 1, *P* = 0.0029, Additional file 4: Figure S3). We found that unfiltered biofilm and biofilm supernatant of all species caused significant mortality over seven days, with most of the mortality induced in the first 72 h (Fig. 7). Biofilm supernatant from *C.*sp_P had the strongest mosquitocidal activity, causing 100% mortality by 48 h (Fig. 7a). Significant mosquitocidal activity was retained in the cell-free filtrates of *C.*sp_P biofilm supernatant (Fig. 7a), *C. subtsugae* biofilm supernatant (Fig. 7c), and *C. vaccinii* biofilm (Fig. 7e). Removing live cells by filtration eliminated all mosquitocidal activity from *C. aquaticum* (Fig. 7b) and *C. violaceum* (Fig. 7d).

## Discussion

Exposure to *C.*sp_P causes significant mortality in the disease vector mosquitoes *An. gambiae* and *Ae. aegypti* [17]. In this study, we aimed to further elucidate the effects of the bacteria on vector mosquitoes. Our first goal was to assess whether exposing females to a sub-lethal dose of *C.*sp_P had any effects on their fecundity, fertility, or survival among their F1 offspring. We therefore blood-fed *C.*sp_P*-* or PBS-exposed females, collected their eggs, and reared F1 offspring to evaluate transgenerational fitness effects. We found that exposure of adult female *An. gambiae* to *C.*sp_P had no effect on probability of oviposition, average number of eggs laid, or the percentage of eggs that hatched. However, maternal exposure to *C.*sp_P was associated with increased larval mortality, delayed pupation, and delayed eclosion of F1 offspring. Maternal *C.*sp_P exposure had no effect on the longevity of F1 adult offspring. These results suggest that sub-lethal *C.*sp_P exposure of adult females causes transgenerational effects on their offspring during immature stages of development.

Bacteria present in adult females have been found on the surface of mosquito eggs [9], and it is therefore possible that *C.*sp_P may have been vertically transmitted to larval offspring by egg smearing or that *C.*sp_P could have been present in the larval breeding water throughout development. *C.*sp_P was not detected in oviposition water and was only detected in larval water in a single measurement from one of three replicates, suggesting that *C.*sp_P was generally not present in oviposition or breeding water of F1 larvae. It is possible that *C.*sp_P was present transiently or in low levels that we failed to detect, and we therefore cannot exclude that *C.*sp_P directly influenced larval mortality and developmental delays *via* vertical transmission or environmental exposure. However, the transgenerational fitness effects were consistent across replicates regardless of whether *C.*sp_P was detected in larval water, suggesting these phenotypes may be attributable to indirect effects of maternal bacterial exposure.

Exposure to various pathogens has been shown either to decrease (e.g. [37–41]) or to not affect (e.g. [42, 43]) fecundity in mosquitoes, but the effects of parental bacterial exposure on developmental time and longevity of mosquito offspring remain unclear. In *Drosophila melanogaster*, infection of females with bacteria has been shown to cause reduced longevity of offspring, though these effects varied between genetic lines [44]. In *Tenebrio molitor*, induction of an immune response in females has been shown to cause longer development time in their larval offspring, and in *Trichoplusia ni*, parental dietary exposure to bacteria caused decreased survival and prolonged development time in offspring [45, 46]. Exposure to *C.*sp_P elicits an immune response in adult *An. gambiae* [17]. It is possible that the increased larval mortality and delayed development we observed in offspring of *C.*sp_P-exposed mothers is a transgenerational cost of pathogenic infection or of mounting an immune response.

In investigating the effects of exposure to cell-free *C.*sp_P biofilm supernatant on the adult female transcriptome, we found that genes involved in detoxification and response to insecticide exposure were significantly enriched. Exposure to insecticides has been shown to have intragenerational and transgenerational effects in mosquitoes and other insects, though the nature of these effects is diverse. For example, exposure of *An. stephensi* larvae to organophosphate and carbamate insecticides caused decreased fertility but increased longevity in adults [47]. Treatment of *Ae. aegypti* larvae with pyrethroid and organophosphate insecticides as well as a botanical extract shortened development time, increased pupal mortality and decreased adult emergence [48], and treatment of *C. quinquefasciatus* larvae with organophosphates lengthened development time and reduced fecundity [49]. Several studies have documented transgenerational fitness costs of insecticide exposure in other insects. For example, offspring of the *Spodoptera litura* moth treated with methoxyfenozide had lower larval weight and longer development time [50], and exposure of adult thrips (*Scolothrips longicornis*) to insecticide reduced the longevity and fecundity of their offspring [51]. Exposure of adult *Aphis gossypii* aphids to sulfoxaflor caused longer development time in offspring [52], and exposure to the neonicotinoid pesticide cycloxaprid caused shorter development time and reduced longevity and fecundity in offspring [53]. The insects in these studies are diverse, as are the insecticide classes investigated, but this work overall shows that exposure to xenobiotics in one developmental stage can have substantial effects on later developmental stages and even transgenerational effects. *C.*sp_P may produce an insecticide-like compound that is acting to induce fitness costs in the offspring of exposed females.

Given the effects *C.*sp_P has on adult longevity, we were interested in determining whether the mosquitocidal activity of *C.*sp_P could be isolated in cell-free preparations, i.e. independent of live bacteria. This would be valuable from a mosquito control perspective, as bioactive factors that can be isolated and used independent of living microorganisms would be more amenable to formulation and distribution and would prevent the uncertainty and risk of introducing a live microorganism into the environment. We found that treatment of adult *An. gambiae* females with bacterial culture suspension induced substantial mortality, regardless of the age of the culture or whether it was grown in planktonic or biofilm conditions. When we removed live bacterial cells from each preparation by filtering through a 0.2 μm filter, we found that mosquitocidal activity was lost for all preparations except the biofilm supernatant (i.e. the media in which the biofilm was cultured). Biofilms are aggregates of bacterial cells embedded in a complex three-dimensional matrix that form as a result of density dependent cell-to-cell signaling called quorum sensing [54–56]. Quorum sensing and biofilm growth has been linked to secretion of virulence factors in other systems [57, 58]. It is possible that the *C.*sp_P-secreted mosquitocidal factor is produced under all conditions but only secreted under biofilm conditions. Another possibility is that there are multiple factors, some of which are produced regardless of how the bacteria are cultured and some which are produced and secreted only by *C.*sp_P biofilm.

The broad-spectrum poison hydrogen cyanide (HCN) is produced by members of the *Chromobacterium* genus and is regulated in part by quorum sensing [21, 35, 59]. As such, it is a clear candidate for causing *C.*sp_P-induced mosquito mortality. We found that cell-free *C.*sp_P biofilm supernatant does produce hydrogen cyanide at approximately 0.039 mg/l. This means that the 1 ml sugar meal provided to adult females contained a total of approximately 0.02 μg HCN, of which only a fraction would be ingested by each individual mosquito. With the exception of insects that feed on cyanogenic plant species (and have therefore evolved extremely high tolerance to cyanide) [60–62], there is very little information available regarding the susceptibility of terrestrial invertebrates to ingestion of cyanide. Fumigation studies of the wheat weevil, *Sitophilus granarius*, showed that exposure to 28.6 mg/l HCN induced LC50 after an 8 min exposure, and 8 mg/l HCN induced LC50 after 4 h [63, 64]. Given that these levels are orders of magnitude higher than the concentration of HCN in *C.*sp_P biofilm, we consider it unlikely that HCN is the source of mosquitocidal activity. Consistent with this, we found that *C.*sp_P biofilm supernatant caused significant mortality compared to the LB control regardless of whether it was vacuum centrifuged. Were HCN the cause of mosquito mortality, we would have expected it (and the mosquitocidal activity) to be lost after vacuum centrifugation, given that HCN is a gas and boils close to room temperature (25.6 °C). Interestingly, vacuum centrifugation itself caused a significant reduction in survival that was consistent in both treatments (*C.*sp_P and LB control), suggesting the presence of volatile compounds in LB that improve longevity of the adult mosquito.

In addition to exploring the nature of the mosquitocidal activity, we also investigated how exposure to *C.*sp_P impacts the transcriptome of adult female *An. gambiae*. We found that genes related to xenobiotic and insecticide detoxification were upregulated after oral exposure to cell-free *C.*sp_P biofilm supernatant. These genes included many *cytochrome P450* genes, specifically those in the CYP6M, CYP6P, CYP6Y and CYP6Z subfamilies. Genes from the CYP6M, CYP6P and CYP6Z subfamilies have all been shown to play a role in metabolism of (and resistance to) multiple classes of insecticides [65–67]. These findings suggest that the *C.*sp_P biofilm supernatant contains compound(s) that evoke a physiological response in the mosquito similar to that mounted in response to insecticide exposure. It will be valuable in the future to further investigate the nature of the mosquitocidal compounds produced by *C.*sp_P to determine whether they differ from known classes of insecticides.

We also found that exposure to cell-free *C.*sp_P biofilm supernatant resulted in changes in transcript abundance in genes involved in nucleosome and chromatin formation. Specifically, multiple genes that encode for histone proteins were downregulated. Histone proteins form multimeric complexes, around which DNA is wound to form nucleosomes, which then pack together to form chromatin. Histone gene mRNA levels decrease naturally following DNA replication and artificially after treatment with ionizing radiation or drugs that cause DNA damage or stalled DNA replication [68–70]. This suggests that *C.*sp_P may produce a factor that induces this state in the mosquito midgut.

In testing other *Chromobacterium* species, we found that all five tested had significant mosquitocidal activity when live bacterial preparations were fed to adult female *An. gambiae*. After removal of live bacterial cells, *C.*sp_P, *C. subtsugae* and *C. vaccinii* retained mosquitocidal activity while *C. violaceum* and *C. aquaticum* did not. However, the mosquitocidal activity produced by *C.*sp_P was strongest among all species tested, both before and after removal of live cells. One possible interpretation of these results is that mosquitocidal factor(s) are produced by all members of the genus, but only secreted in lethal concentrations by *C.*sp_P, *C. subtsugae* and *C. vaccinii*. *C.*sp_P’s especially robust activity may be due to high production of these mosquitocidal factors, or production of a unique factor not made by the other species in the genus. *Chromobacterium violaceum* and *C. aquaticum* may produce a different mosquitocidal compound that is not secreted, or accumulation in the media of the mosquitocidal factor(s) may be temporally dynamic and we may have failed to detect it in our experiment*.* Alternatively, live *C. violaceum* and *C. aquaticum* may kill mosquitoes by causing a lethal infection. *Chromobacterium subtsugae* is known to have insecticidal activity and is currently being used as a biopesticide marketed as Grandevo® (Marrone Bio Innovations) [20, 36]. *Chromobacterium vaccinii* has also been shown to cause mortality in moths and mosquito larvae, though the mechanism by which either of these species cause insect mortality is currently unknown [21]. Our data also show that, in addition to causing mortality in *An. gambiae* and *Ae. aegypti* [17], *C.*sp_P is active against *An. stephensi* (a major vector of *Plasmodium* in Asia), *Ae. albopictus* (a vector of dengue, Zika, and chikungunya viruses), and *Culex quinquefasciatus* (a vector of West Nile virus). This suggests that the mosquitocidal factor produced by *C.*sp_P generally affects mosquitoes and could be potentially used against a diverse range of mosquito species and possibly other types of insects. *Chromobacterium subtsugae* is very broad in its insecticidal effects, causing mortality in beetles, moths, stinkbugs and whiteflies [20]. Interestingly, *C. subtsugae* does not cause mortality in *Culex pipiens* mosquito larvae, while *C. vaccinii* and *C.*sp_P do cause mortality in *Aedes aegypti* mosquito larvae [17, 20, 21]. These data suggest there may be multiple compounds produced by these species that have the potential for broad-spectrum mosquitocidal activity.

## Conclusions

Our results show that oral exposure to *C.*sp_P induces significant mortality in a broad range of disease vector mosquitoes, and non-lethal exposure of adult females causes increased mortality and slower development in F1 offspring. *Chromobacteria* are known to produce hydrogen cyanide, but our data suggest that this is not the cause of *C.*sp_P-induced mosquito mortality. Mosquitocidal activity persists after removal of live bacterial cells from *C.*sp_P biofilm culture media, and oral exposure to this *C.*sp_P treated media elicits changes in the mosquito midgut transcriptome that are similar to those that occur after exposure to insecticidal compounds and other xenobiotics. Finally, other *Chromobacterium* species also cause increased mortality in adult *An. gambiae* suggesting the *Chromobacterium* genus holds potential for the exploration for novel mosquitocidal compounds. That the mosquitocidal factor(s) can be isolated in cell-free preparations renders them amenable to further biochemical study to determine mode of action and increases their potential for use as chemical insecticides.

## Additional files

Additional file 1: **Figure S1.** Proportion of inseminated females exposed to each bacterial treatment. Insemination status of females from each group was assessed and found to not differ. Data were collected over 3–4 replicates. Effect of treatment on insemination status was evaluated using a Kruskal Wallis test in R (*χ*^2^ = 4.77, *df* = 2, *P* = 0.092). (TIFF 208 kb)

Additional file 2: **Figure S2.** Treatment of adult *An. gambiae* females with *C.*sp_P does not result in increased bacterial load in breeding water of larval offspring. Two 1 ml water samples were taken from the oviposition cups and from larval trays prior to adding food or larvae (baseline), and then again at 4 and 8 days after transfer of the larvae. Although the load of cultivable bacteria differed across time (*F*_(3, 19)_ = 388.06, *df* = 3, *P* <0.0001), the mean bacterial load was not significantly different between the two groups *F*_(1, 19)_ = 0.04, *df* = 1, *P* = 0.842 and this was consistent across time (time × treatment interaction: *F*_(3, 16)_ = 0.13, *df* = 3, *P* = 0.944). Each data point represents the average CFU of cultivable bacteria for each of the three experimental replicates; error bars represent 95% confidence intervals. A two-way ANOVA was used to analyze the data. (TIFF 245 kb)

Additional file 3: Raw data for Figs. 1–7. (XLSX 185 kb)

Additional file 4: **Figure S3.** *Chromobacterium* species biofilm harbors more bacteria than supernatant. Each species was cultured under biofilm conditions and CFU/ml were estimated from biofilm and biofilm supernatant fractions of each species. A two-factor ANOVA including species and culture fraction as factors revealed no interaction between the factors (*F*_(4, 18)_ = 1.08, *df* = 4, *P* = 0.394), and there was a significant main effect of culture fraction (*F*_(1, 22)_ = 11.26, *df* = 1, *P* = 0.0029) but not of species (*F*_(4, 22)_ = 0.23, *df* = 4, *P* = 0.92). (TIFF 369 kb)

## Abbreviations

*C.*sp_P: *Chromobacterium* species Panama
PBS: phosphate-buffered saline
BEI Resources: Biodefense and Emerging Infections Research Resources Repository
ATCC: American Type Culture Collection
DSMZ: Deutsche Sammlung von Mikroorganismen und Zellkulturen
LB: lysogeny broth
CFU: Colony Forming Units
HCN: hydrogen cyanide
