# Supplementary figures and images for: The mosquito adulticidal Chromobacterium sp. Panama causes transgenerational impacts on fitness parameters and elicits xenobiotic gene responses
Source: Parasit Vectors. 2018 Apr 5;11:229. doi: 10.1186/s13071-018-2822-8 (PMC5887189; doi:10.1186/s13071-018-2822-8)

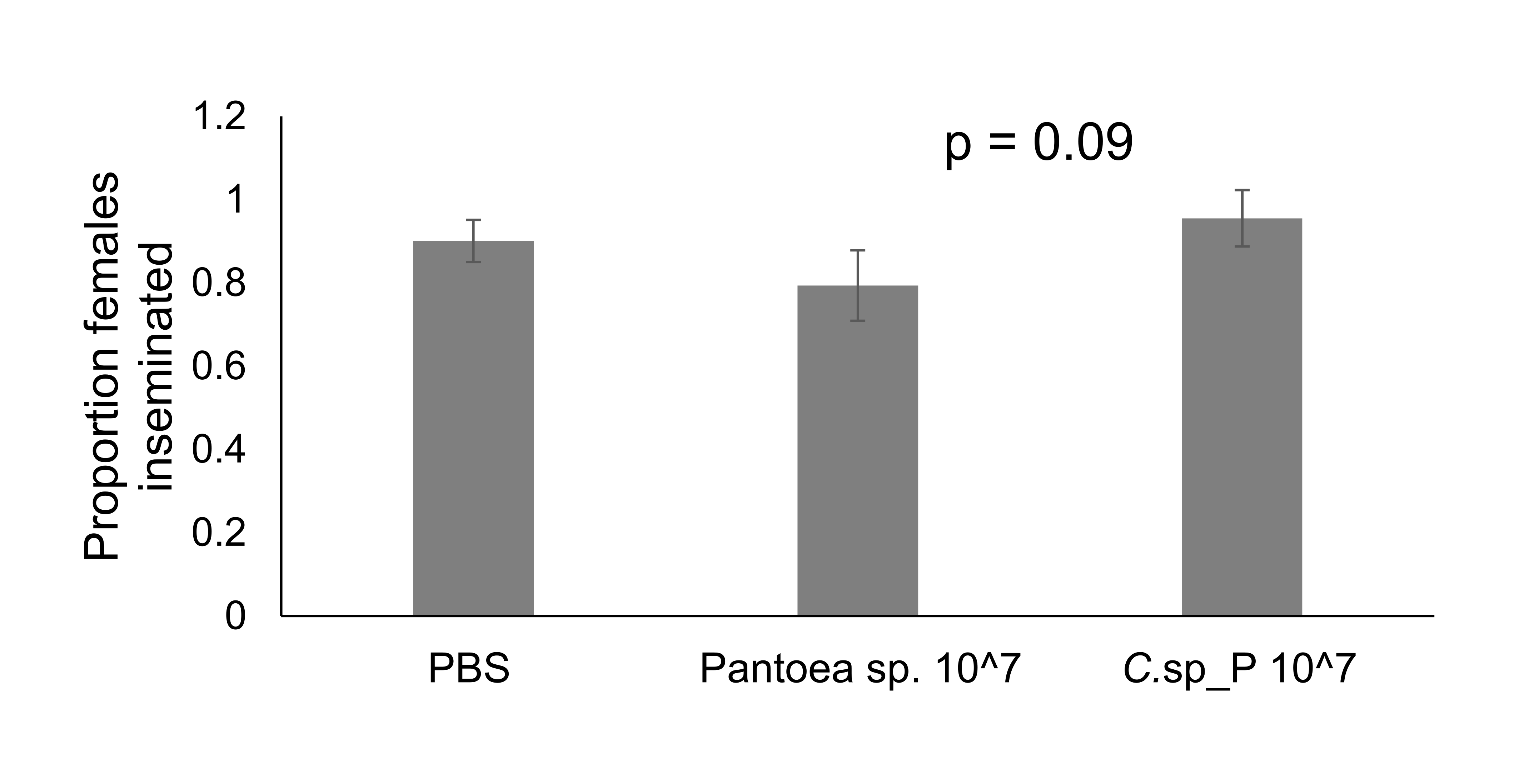

Supplement: Supplementary file 1 — Figure S1. Proportion of inseminated females exposed to each bacterial treatment. Insemination status of females from each group was assessed and found to not differ. Data were collected over 3–4 replicates. Effect of treatment on insemination status was evaluated using a Kruskal Wallis test in R (χ2 = 4.77, df = 2, P = 0.092). (TIFF 208 kb) [file 13071_2018_2822_MOESM1_ESM.tif]

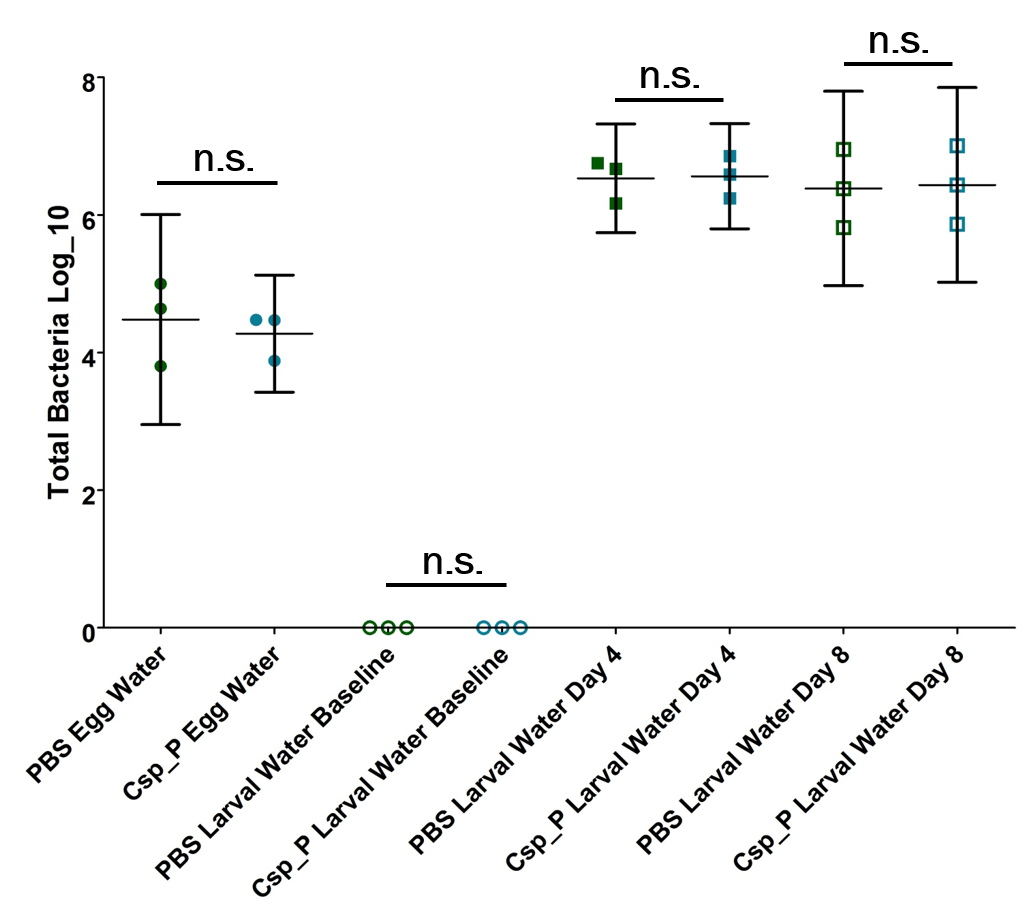

Supplement: Supplementary file 2 — Figure S2. Treatment of adult An. gambiae females with C.sp_P does not result in increased bacterial load in breeding water of larval offspring. Two 1 ml water samples were taken from the oviposition cups and from larval trays prior to adding food or larvae (baseline), and then again at 4 and 8 days after transfer of the larvae. Although the load of cultivable bacteria differed across time (F(3, 19) = 388.06, df = 3, P <0.0001), the mean bacterial load was not significantly different between the two groups F(1, 19) = 0.04, df = 1, P = 0.842 and this was consistent across time (time × treatment interaction: F(3, 16) = 0.13, df = 3, P = 0.944). Each data point represents the average CFU of cultivable bacteria for each of the three experimental replicates; error bars represent 95% confidence intervals. A two-way ANOVA was used to analyze the data. (TIFF 245 kb) [file 13071_2018_2822_MOESM2_ESM.tif]

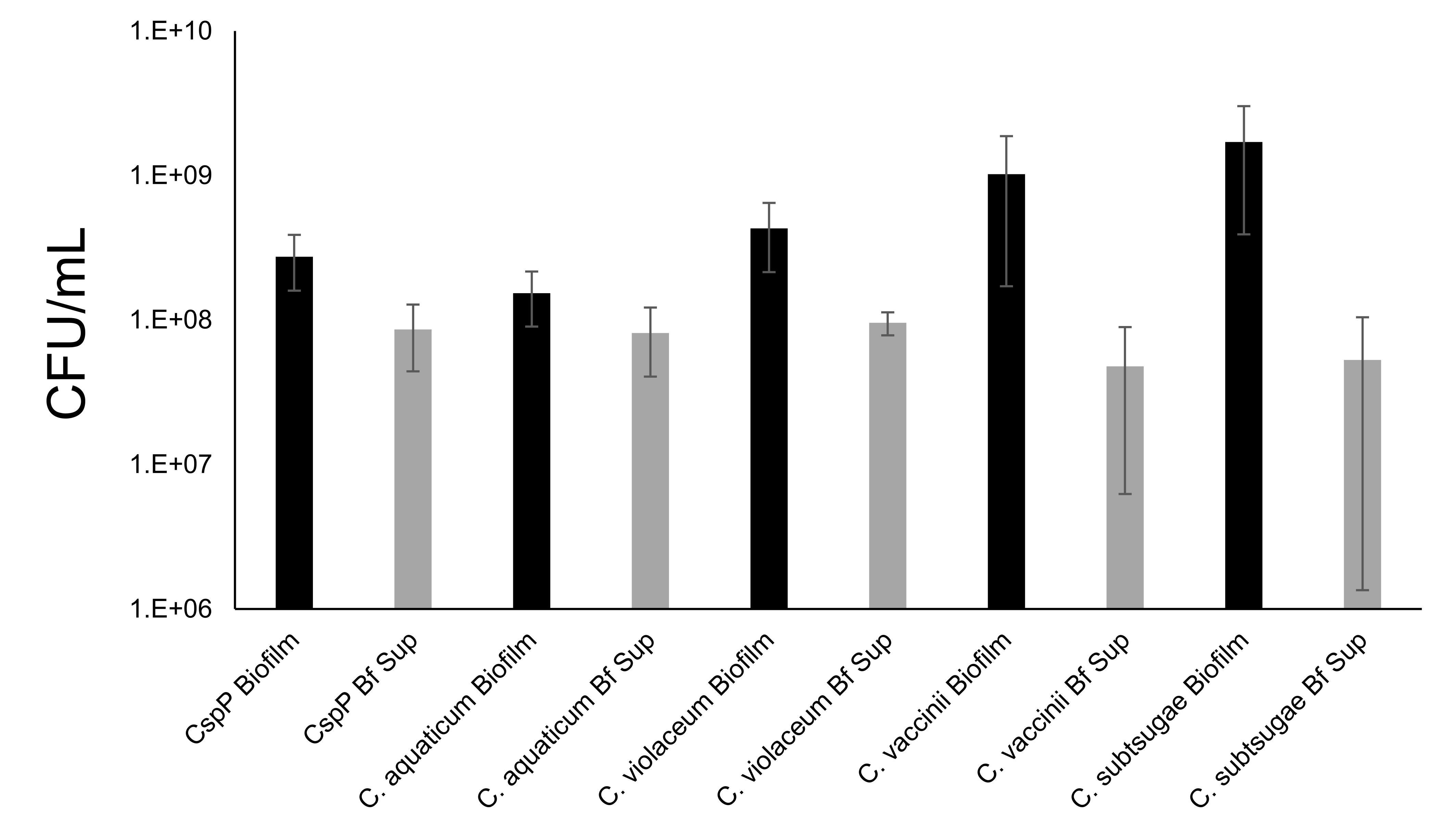

Supplement: Supplementary file 4 — Figure S3. Chromobacterium species biofilm harbors more bacteria than supernatant. Each species was cultured under biofilm conditions and CFU/ml were estimated from biofilm and biofilm supernatant fractions of each species. A two-factor ANOVA including species and culture fraction as factors revealed no interaction between the factors (F(4, 18) = 1.08, df = 4, P = 0.394), and there was a significant main effect of culture fraction (F(1, 22) = 11.26, df = 1, P = 0.0029) but not of species (F(4, 22) = 0.23, df = 4, P = 0.92). (TIFF 369 kb) [file 13071_2018_2822_MOESM4_ESM.tif]
